# Needle-free vascular access port for hemodialysis: Proof-of-concept validation in an experimental animal study

**DOI:** 10.1016/j.jvscit.2025.101983

**Published:** 2025-09-13

**Authors:** Bernard Canaud, Abdessalem Hammed, Alexandre Karsenti, Ludovic Canaud, Eric Jean, Jean-Marie Vrinat, Hafedh Fessi, Jean-Yves Ayoub, Michael Rys, Vanessa Louzier

**Affiliations:** a Faculty of Medicine, University of Montpellier, Montpellier, France; b UR APCSe Agressions Pulmonaires et Circulatoires Dans le Sepsis, VetAgro Sup, Université de Lyon, Marcy l'Etoile, France; c Thoracic and Cardiovascular Surgery Department, Marne La Vallée Hospital, GHEF, Marne La Vallée, France; d Vascular and Thoracic Surgery Department, Arnaud de Villeneuve CHI Montpellier, Montpellier, France; e SÆPTUM SAS, Hérouville-Saint-Clair, France; f R&D Department, Meditor SAS, La Wantzenau, France; g Nephrology Department, Tenon Hospital, Paris, France; h Unité de Physiologie, Pharmacodynamie et Thérapeutique, VetAgro Sup, Université de Lyon, Marcy l'Etoile, France

**Keywords:** Vascular access for hemodialysis, Arteriovenous port system, Hemodialysis, Needle-free access

## Abstract

Vascular access remains a critical challenge in kidney replacement therapy. Although arteriovenous fistulas are the reference standard, they are often compromised by maturation failure, complications, and repeated needling, which causes pain and distress for patients. A needle-free alternative could enhance safety, simplify use, and improve the patient experience, particularly in self-care and home dialysis. We evaluated the Safe Hemodialysis Implantable Vascular Access Technology arteriovenous port, a patented titanium device incorporating a twist-lock system anastomosed to a vascular graft prosthesis. The proof-of-concept study included in vitro testing to assess flow performance, durability, and hemocompatibility, followed by implantation in two sheep to determine surgical feasibility, hemodynamic function, and safety during a sham hemodialysis session. Implantation was successful without complications, the twist-lock system functioned securely, and no blood leakage, arterial bleeding, or air embolism occurred. The device provided adequate blood flow, although refinements are required to achieve clinical targets of 300–400 mL/min. These preliminary findings suggest that the Safe Hemodialysis Implantable Vascular Access Technology port is a feasible and safe needle-free vascular access concept, with potential to reduce needling burden, improve handling, and minimize infection risk in clinical use.

Vascular access is a critical component of extracorporeal kidney replacement therapy for end-stage kidney disease patients. However, it remains one of the most challenging aspects of treatment. The native arteriovenous fistula (AVF) is widely considered the gold standard for permanent vascular access owing to its superior performance and lower morbidity when properly created and managed.^1^

Despite its advantages, the AVF is not without limitations. It is not always feasible for implantation, may fail to mature, can be associated with dysfunction, and can develop complications over time.^2^, ^3^, ^4^, ^5^, ^6^ Additionally, the needling process is a significant source of distress and fear for patients owing to pain and anxiety, particularly in the context of self-care and home hemodialysis, where frequent cannulation is required for daily or intensified treatment regimens.^7^, ^8^, ^9^, ^10^ In this context, the recent Kidney Disease Outcomes Quality Initiative guidelines on vascular access management recommend an individualized, patient-centered approach to vascular access planning, emphasizing the End-Stage Kidney Disease Life-Plan, which optimizes access selection, minimizes complications, and ensures long-term dialysis success.^11^

In recent years, innovation in vascular access for extracorporeal therapy has led to the development of advanced techniques and vascular grafts to facilitate vascular access creation, although not necessarily prioritizing needle-free solutions or patient-centered perspectives.^12^ Although tunneled central venous catheters remain widely used, they are associated with high risks of infection, stenosis, thrombosis, and dysfunction, despite the broader use of specific locking solutions. Meanwhile, venous port catheter systems such as Dialock and Lifesite,^13^^,^^14^ as well as arteriovenous port systems like Hemasite,^15^, ^16^, ^17^, ^18^, ^19^ have largely been abandoned owing to complications and cost concerns.

In this context, there is a strong need for a novel, implantable vascular access port that leverages an arteriovenous shunt with a needle-free system, offering greater patient comfort, reliability, and ease of use.

This preliminary study presents the engineering development and the first experimental animal evaluation to validate the proof of concept of this vascular access port. We introduce the Safe Hemodialysis Implantable Vascular Access Technology (SHIVAT), a next-generation vascular access port, detailing its design evolution and its first in vivo validation in an animal model.

## Methods

### SHIVAT vascular access port concept

**Fig 1:**
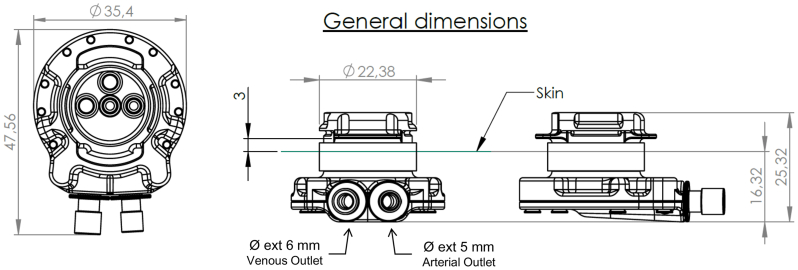
The Safe Hemodialysis Implantable Vascular Access Technology (SHIVAT) port is a compact implantable system (25 mm, 43 g) with a circular titanium body, a central blood flow canal, and two access points for arterial and venous graft connections. Device dimensions are as follows: venous outlet, Ø 6 mm; arterial inlet, Ø 5 mm; internal orifices, Ø 4.71 mm; and rotor connector, Ø 3.2 mm.

SHIVAT is a compact (25 mm), surgically implanted, transcutaneous vascular port access device designed for extracorporeal therapies, including hemodialysis, hemodiafiltration, and apheresis.^20^^,^^21^ The device integrates the patented Hemoplug smart valving system and a dual-lumen internal conduit to establish a direct connection between artery and vein. The SHIVAT port system (Fig 1) consists of a circular metallic main body, made of medical-grade titanium. It incorporates a central blood flow canal connected to two port outlets for arterial and venous access. These outlets can be fitted with vascular grafts (eg, polytetrafluoroethylene [PTFE]) either preassembled by the manufacturer or connected intraoperatively at the time of surgical insertion, thereby enabling seamless integration with the recipient's vasculature. The device weighs 43 g and its dimensions are as follows: external diameter of the venous outlet (graft connection), 6 mm; external diameter of the arterial inlet (graft connection), 5 mm; internal diameter of the upper orifices connected to the arterial and venous lines, Ø 4.71 mm; and internal diameter of the connector plugged into the rotor, Ø 3.2 mm.

**Fig 2:**
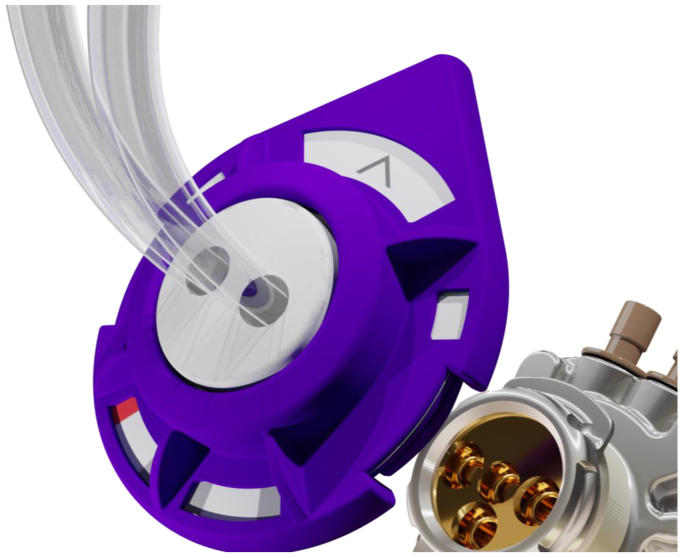
The front face of the Safe Hemodialysis Implantable Vascular Access Technology (SHIVAT) port features a twist-lock connection interface designed for docking with a single-use disposable polymer plug. The plug includes arterial and venous extension lines, an intravenous priming line, and multiple channel positions to allow versatile use.

**Fig 3:**
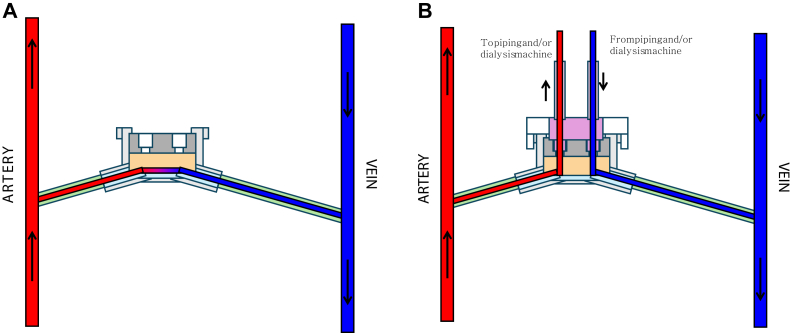
Twist-lock system with disposable plug securely attached to the Safe Hemodialysis Implantable Vascular Access Technology (SHIVAT) port. The plug includes extension tubing for arterial and venous connections and an intravenous priming line. A quarter turn rotation allows channel selection: **(A)** open position for blood flow through the device and **(B)** closed position for patency checks or hemodialysis treatment.

On its front face, the SHIVAT port features a dedicated connection interface designed for the docking of a proprietary medical grade polymer, single-use disposable circular plug (Fig 2). This twist-lock system securely attaches to the port and includes extension tubing for arterial and venous connections, as well as an intravenous priming line. The twist-lock disposable plug offers multiple channel positions for enhanced versatility (Fig 3). With a simple quarter-turn rotation, users can selectively open specific channels, directing blood to either flow through the device (Fig 3, *A*, open) or be redirected into the disposable kit for patency checks or hemodialysis treatment (Fig 3, *B*, closed).

Notably, once the twist-lock disposable plug is attached to the port, the system remains closed and secured, eliminating the need for disconnections or multiple handling steps. Operation is performed through a simple quarter-turn selection mechanism, ensuring a safe, efficient, and user-friendly experience for both patients and health care providers.

The twist-lock port must be integrated with a vascular graft system, either PTFE or another vascular prosthesis, connected to the patient's vasculature. The intended function is to enable a secure, needle-free connection to an extracorporeal circuit through a proprietary twist-lock mechanism, with the vascular graft serving as the permanent conduit to the patient's circulation. The device is adaptable to multiple anatomical implantation sites, including the upper arm, forearm, or inguinal region, allowing flexibility based on clinical and anatomical considerations. Owing to its needle-free design and simplified handling, the device is also intended, in future developments, for potential use in self-care or home-based dialysis treatment by appropriately trained patients. This indication remains investigational and has not yet undergone clinical validation.

### From bench to animal testing: In vitro experimental study

A series of in vitro bench tests were conducted on the SHIVAT port device to evaluate its safety, hemodynamic performance, and structural integrity before animal testing. A custom-built flow test bench was developed to replicate the human circulatory system under physiological conditions, using a temperature-controlled environment maintained at 37°C and circulating fresh pig blood as the testing medium. The primary objectives were to ensure a maximal blood flow rate of 500 mL/min without flow restriction, to confirm that the bypass flow in the closed position remained below 1000 mL/min, and to assess the device's mechanical durability under repeated use.

Bench testing was performed to assess the safety of the SHIVAT port device and presented in Supplementary Figs 1 and 2 (online only). Rotational friction refers to the resistance encountered when one titanium plate rotates against another static titanium plate. In this medical device, friction was tested before device sterilization to confirm smooth, consistent rotation without excessive effort or irregularities. This step ensures compliance, reliability, and proper mechanical function of the device. Sealing integrity was assessed by applying a safety coefficient of 1.5 to pressures exceeding both physiological and device-related conditions. The evaluation considered average arterial pressure (150 mm Hg) as well as extreme negative and positive pressures generated by the dialysis machine (−300 mm Hg to +500 mm Hg), thereby ensuring robust sealing performance under all expected operating conditions. Biocompatibility results were derived from prior validated testing of a similar CE-marked device (UPLUG), in compliance with ISO 10993. According to ISO 10993-4, the device demonstrated no in vitro human complement activation, no hemolysis under direct blood contact, and no activation of the coagulation contact phase after 15 minutes of direct exposure to human platelet-poor plasma. Bioburden was within acceptable limits (<100 colony-forming units [CFU]). Average values measured were less than 5 CFU for the port and less than 20 CFU for the disposable component. An additional schematic description of the bench testing has been added.

All components in contact with circulating blood, specifically the rotor and inlay, were compliant with ISO 10993 biocompatibility standards, consistent with those used in the approved UPLUG device. The stator and intermediate disk were manufactured from medical-grade polyether ether ketone, known for its mechanical resilience and chemical stability in clinical settings.

To release the two subject devices for further evaluation, specific functional tests were conducted. Each device underwent 10 successive rotation cycles to verify that rotational friction remained stable and that no gripping or sticking phenomena occurred. Sealing integrity was also tested under pressure conditions simulating clinical use: in the closed (bypass) position, the device was subjected to a positive pressure of +220 mm Hg, and in the open (extracorporeal circulation) position, pressures of +750 mm Hg and −500 mm Hg were applied to evaluate performance under both positive and negative gradients. Finally, the devices were sterilized using beta radiation to ensure microbiological safety and compliance with standard sterilization protocols for implantable medical devices. All components of the SHIVAT vascular port access are listed in a table provided in the Supplementary Table (online only).

### Animal study

This study was conducted in accordance with the Guide for the Care and Use of Laboratory Animals. All animal procedures were approved by the Ethics Committee of our institution (VetAgro Sup; authorization number: 2150) and were carried out in compliance with Directive 2010/63/EU of the European Parliament on the protection of animals used for scientific purposes. This article was prepared in accordance with the ARRIVE 2.0 guidelines.

The study was structured into two key phases to assess feasibility and performance of the SHIVAT device: (1) Surgical implantation: The SHIVAT device was implanted in two adult sheep, which had been preconditioned for one week before the procedure. (2) Sham hemodialysis sessions: after implantation, sham hemodialysis sessions were conducted to evaluate on conscious sheep to mimic clinical conditions in human dialysis patients and to evaluate the device's functionality under controlled conditions.

The animals were housed in a dedicated, specialized facility designed to ensure their well-being, postoperative recovery, and continuous monitoring throughout the study.

### Surgical implantation of SHIVAT

#### Animals

Two adult male sheep, weighing 73 and 78 kg, were included in the study. An acclimation period of 8 days was observed before the start of the experiment. The animals were fed hay ad libitum, supplemented with alfalfa pellets, and had free access to water. On the day before the experiment, each sheep was anesthetized and surgically prepared. Tranquilization was achieved with an intramuscular injection of morphine (0.2 mg/kg) followed by an intravenous injection of diazepam (0.25 mg/kg). Anesthesia was induced by slow intravenous injection of propofol (4 mg/kg), followed by intubation using an 8.5F endotracheal tube and maintenance with sevoflurane at 2%. The SHIVAT device, designed to establish a connection between the internal carotid artery and the external jugular vein, was implanted on the left side of the neck. The procedure was carried out by two vascular surgeons under the supervision and guidance of a veterinary expert.

#### Preoperative preparation

The sheep's necks were shaved and prepared. The cervical vessels were identified by an ultrasound probe, and skin markings were drawn to determine the precise placement of the SHIVAT device and vascular grafts. The selected skin area was disinfected with povidone-iodine (Betadine) before surgery. A sterile surgical drape was positioned to protect the operative field.

#### Surgical procedure

**Fig 4:**
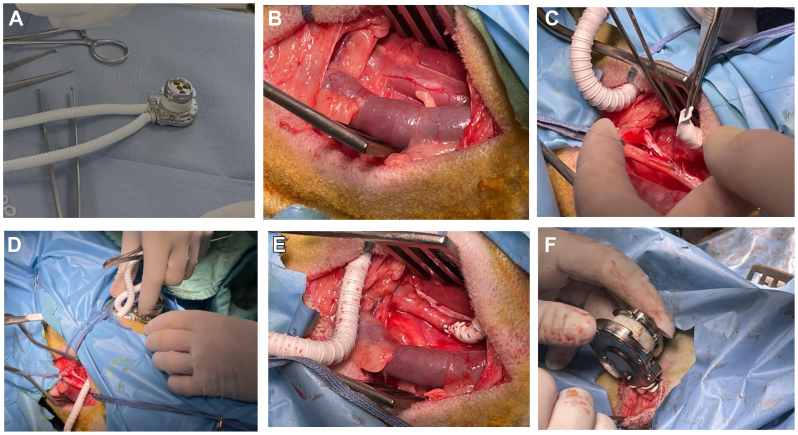
Main steps of Safe Hemodialysis Implantable Vascular Access Technology (SHIVAT) surgical implantation. **(A)** Device preparation. The SHIVAT port, preassembled with polytetrafluoroethylene (PTFE) vascular grafts, is prepared on a sterile field. **(B)** Vessel exposure. Surgical dissection and exposure of the target artery and vein. **(C)** Arteriotomy and venotomy. The artery and vein are isolated and clamped and sites are prepared for anastomosis. **(D)** Graft anastomosis. PTFE grafts are sutured to the artery and vein, ensuring secure inflow (arterial) and outflow (venous) connections. **(E)** Graft orientation check. Verification of graft positioning and patency after anastomosis. **(F)** Port implantation. The SHIVAT device is inserted into a subcutaneous pocket and connected to the grafts.

**Fig 5:**
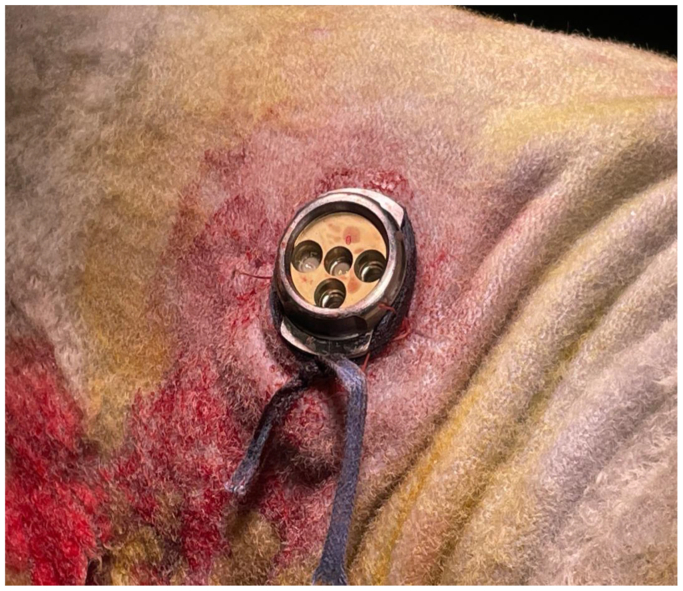
A circular skin opening was created to externalize the connecting face of the Safe Hemodialysis Implantable Vascular Access Technology (SHIVAT) port, aligning precisely with the diameter of the device.

**Fig 6:**
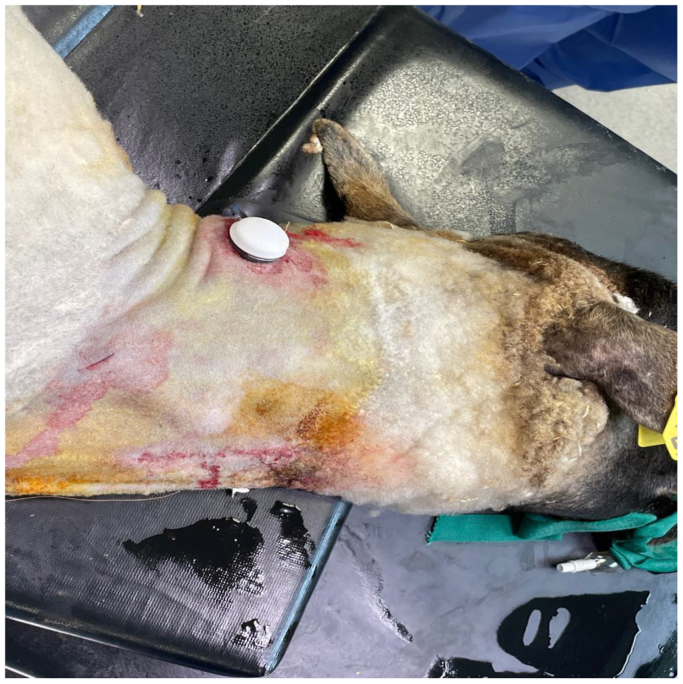
A protective cap is placed over the Safe Hemodialysis Implantable Vascular Access Technology (SHIVAT) port to ensure secure closure and protection when the port is not in use.

Fig 4 summarizes the main steps of SHIVAT surgical implantation. Before implantation, the SHIVAT device was fitted with a PTFE graft and tightly sutured, then arterial and venous graft branches were flushed, filled with isotonic saline, and clamped. After making a skin incision, the internal carotid artery and external jugular vein were dissected carefully, isolated, and secured with two vascular loops to facilitate vascular incision. The arterial graft was anastomosed to the internal carotid artery using a side-to-end vascular suture, and the venous graft was anastomosed to the external jugular vein using the same technique. A small circular skin opening, matching the diameter of the SHIVAT device, was created on the neck to externalize the connecting face of the SHIVAT (Fig 5). After ensuring a leak-free anastomosis, the vascular clamps were released, allowing blood flow through the device. The functionality of the SHIVAT system was then evaluated using the twist-lock disposable connector to verify its patency. The skin incision was then closed in two layers subcutaneously and skin closure ensured with surgical sutures. A protective cap was placed on the SHIVAT device to ensure closure and protection (Fig 6). The skin was cleaned, and a sterile bandage was applied to protect the incision site.

#### Postsurgical care

After the surgical procedure, the sheep were gradually awakened and monitored in their enclosures until they had fully recovered.

### Hemodialysis protocol

On the day of hemodialysis, the two sheep were placed in their own enclosures within the dialysis room to keep them calm. At the end of the session, they were returned to a shared enclosure within the facility. The physiological status of each sheep was monitored before, during, and after dialysis by assessing food and water intake, as well as observing rumination, mobility, and behavior.

**Fig 7:**
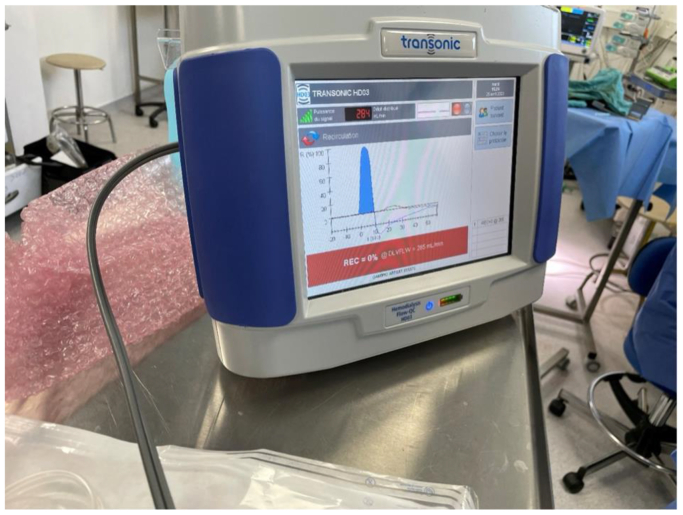
In vivo assessment of Safe Hemodialysis Implantable Vascular Access Technology (SHIVAT) port performance. The blood flow rate and recirculation were measured using a transonic device during dialysis.

Hemodialysis was performed using a Prismaflex monitor for a duration of 1 h, with continuous monitoring of the blood flow rate. SHIVAT flow and pressure were assessed with the hemodialysis monitor, and effective access flow and recirculation were measured using the TRANSONIC device (Fig 7). The dialysate solution used was Hemosol B0, supplemented with potassium to a final concentration of 4.5 mmol/L. Systemic anticoagulation was achieved with standard heparin, administered at a dose of 5000 IU/L to maintain circuit patency during the session.

## Results

The patency of the SHIVAT device was assessed in vivo after insertion in the animal model. Ultrasound imaging was used to evaluate immediate and short-term blood flow through the system before and after connection to the CRRT device during a hemodialysis session. This noninvasive assessment confirmed appropriate positioning and initial functionality of the device.

The usability and functionality of the SHIVAT port were further assessed through sham hemodialysis sessions, ensuring effective vascular access performance under simulated hemodialysis conditions. After the surgery, each sheep underwent two sham hemodialysis sessions using a Prismaflex hemodialysis machine. Each session lasted 60 minutes, with the blood flow rate increased stepwise every 20 minutes from 200 to 300 mL/min. Hemodialysis was performed in isovolemic conditions, and arterial and venous pressures within the extracorporeal circuit were monitored continuously. Notably, the sessions were conducted simultaneously, with both sheep undergoing hemodialysis in the same room at the same time, each animal being kept in their own enclosures, to keep them calm. In addition, intrasession flow performance was monitored using a Transonic flow measurement system (Fig 7). During dialysis, a peak blood flow rate of 285 mL/min was recorded, with no detectable recirculation, as illustrated in the representative screenshot captured during one of the hemodialysis sessions (Fig 6). The hemodialysis procedures were well-tolerated, with no immediate complications observed during the treatment period. At the end of the study, the animals underwent a second surgical procedure for device removal. After recovery, the sheep were returned to their pasture in good health.

## Discussion

The SHIVAT arteriovenous port is an adjunct vascular access device designed for hemodialysis and other extracorporeal blood therapies in patients requiring long-term treatment. Its purpose is to facilitate and secure extracorporeal connection. The port provides a needle-free interface through a proprietary twist-lock mechanism, enabling priming, treatment, and rinse-back through a single closed-loop interface, thereby reducing handling steps and minimizing the risk of contamination. Surgical implantation is required, in combination with a compatible vascular graft (PTFE or equivalent vascular prosthesis) anastomosed to the patient's arterial and venous circulation. Potential implantation sites include the upper arm, forearm, or inguinal region, as determined by clinical assessment. The SHIVAT port is not indicated for direct vessel insertion without a vascular graft, and the use of a graft carries its own inherent risks. Potential applications in self-care or home-based dialysis remain investigational and have not yet received regulatory clearance or clinical validation.

This innovation offers three key theoretical benefits from a clinical perspective. First, it eliminates the repeated needling of an AVF, significantly reducing pain and alleviating the fear associated with needle insertion, an important advantage for patient comfort and adherence to treatment.^7^^,^^8^^,^^10^ Second, it streamlines the connection and disconnection process by removing the complex and potentially hazardous handling associated with clamping and vascular access opening during connection and disconnection of hemodialysis sessions.^5^ This not only improves safety, but also enhances ease of use for health care providers, addressing critical challenges in vascular access management.^22^, ^23^, ^24^ By reducing manual interventions and eliminating open access points, the SHIVAT port minimizes infection risks and enhances the overall efficiency of dialysis procedures. Third, as a needle-free alternative, it offers a user-friendly option for self-care, empowering patients, boosting their confidence, enhancing the treatment experience, and addressing patient advocacy for greater comfort, thereby facilitating home treatment.^25^

In this pilot animal study, we validated the proof-of-concept functionality of the port SHIVAT device for hemodialysis using extracorporeal blood circulation. From a surgical perspective, implantation of the device was found to be straightforward for an experienced vascular surgeon, with no significant technical difficulties encountered. Functionally, the SHIVAT arteriovenous port successfully delivered the necessary blood flow for short-term hemodialysis in this experimental setting. However, in the animal model, it was observed that further refinements in internal diameter, resistance, and flow dynamics may be necessary to achieve the optimal blood flow rate of 300 to 400 mL/min required for human treatment. These adjustments are primarily related to the design and calibration of the internal channel of the device.

Additionally, the study confirmed the safety of the SHIVAT port; no instances of blood leakage from the device were observed. The twist-lock mechanism, operating with a simple quarter-turn motion, proved to be both intuitive and secure, offering a safer and more convenient alternative to traditional vascular access methods, such as needle cannulation of AVFs. Notably, no complications related to arterial pressure-induced bleeding or air embolism were detected, despite the absence of additional clamping systems.

Overall, the results of this pilot study support the feasibility and potential advantages of the SHIVAT port when used with an arteriovenous graft. Although further refinements are required to optimize its use in human applications, its innovative approach to vascular access holds significant promise in reducing patient discomfort, enhancing safety, and simplifying dialysis procedures for both patients and health care providers.

## Conclusions

The SHIVAT port presents a significant advancement when used with an arteriovenous graft for hemodialysis, offering a needle-free, twist-lock system that enhances patient comfort and procedural efficiency. This pilot study demonstrated its feasibility, surgical simplicity, and functional safety in an animal model, with successful blood flow performance and no observed complications. By eliminating needling, potentially reducing infection risks, and simplifying vascular access management, SHIVAT port has the potential to improve hemodialysis patient care. Although further refinements are needed for human application, these findings support its promise as a safer, more patient-friendly alternative to conventional vascular access methods.

## Funding

This work was funded by Uplug SAS, which designed and engineered the vascular access port. The company was involved in the trial design but had no influence on the study findings.

## Disclosures

E.J. and M.R. are employees of Saeptum. J.M.V. is an employee of Meditor.

## Appendix (online only)

Appendix Supplementary Fig 1. Components used in the Safe Hemodialysis Implantable Vascular Access Technology (SHIVAT) vascular port access. Supplementary Fig 2. Bench testing performed to assess the safety and mechanical function of the Safe Hemodialysis Implantable Vascular Access Technology (SHIVAT) port device. Supplementary Table. Additional bench testing, including rotational friction, sealing integrity, and biocompatibility assessment of the Safe Hemodialysis Implantable Vascular Access Technology (SHIVAT) port device
