# Supplementary material for: Needle-free vascular access port for hemodialysis: Proof-of-concept validation in an experimental animal study
Source: J Vasc Surg Cases Innov Tech. 2025 Sep 13;11(6):101983. doi: 10.1016/j.jvscit.2025.101983 (PMC12547820; doi:10.1016/j.jvscit.2025.101983)
Supplement: Appendix — Supplementary Fig 1. Components used in the Safe Hemodialysis Implantable Vascular Access Technology (SHIVAT) vascular port access. Supplementary Fig 2. Bench testing performed to assess the safety and mechanical function of the Safe Hemodialysis Implantable Vascular Access Technology (SHIVAT) port device. Supplementary Table. Additional bench testing, including rotational friction, sealing integrity, and biocompatibility assessment of the Safe Hemodialysis Implantable Vascular Access Technology (SHIVAT) port device [file mmc1.pptx]

## Slide 1
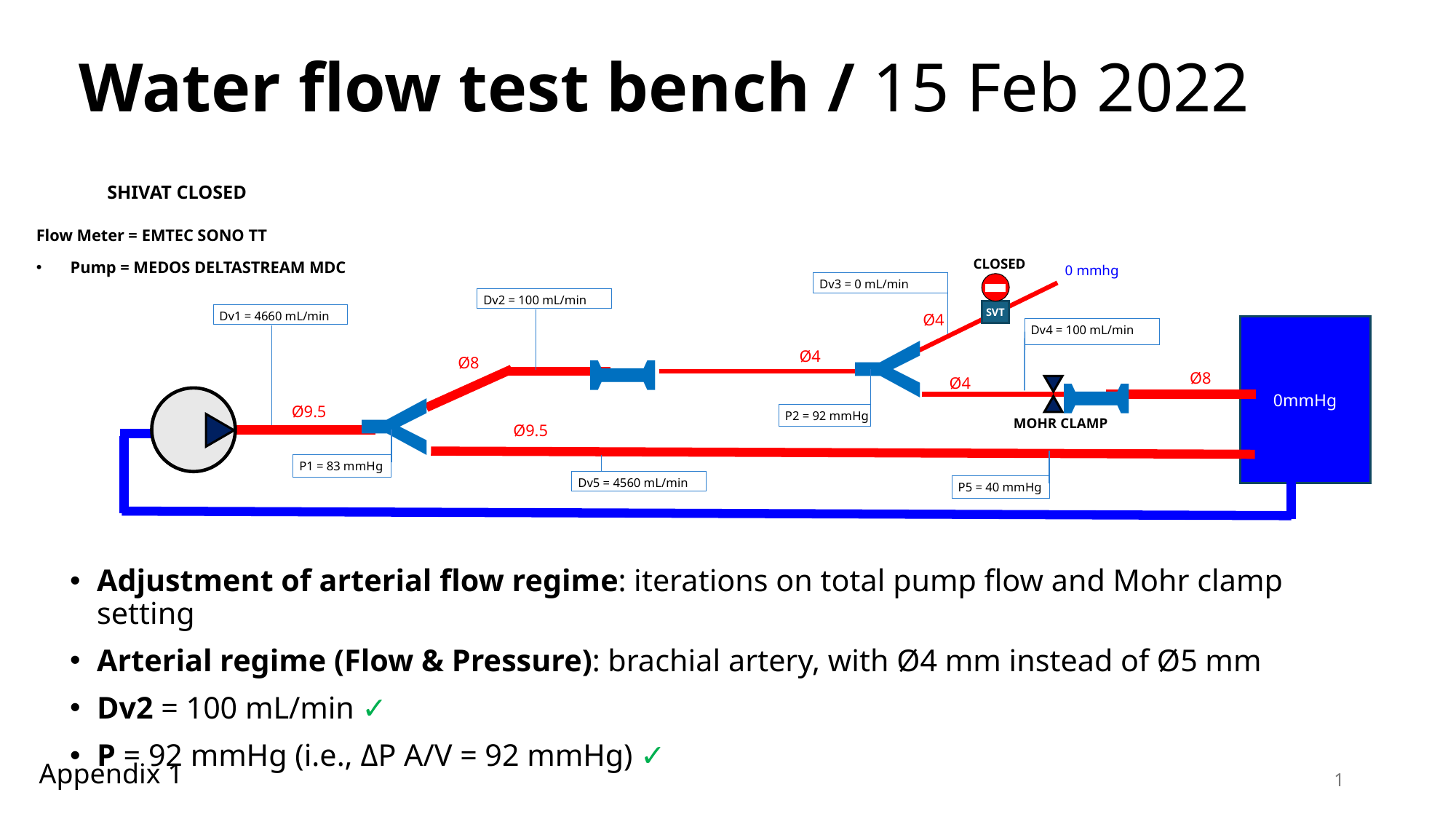

# Water flow test bench / 15 Feb 2022
SHIVAT CLOSED
Flow Meter = EMTEC SONO TT
Pump = MEDOS DELTASTREAM MDC
CLOSED
0 mmhg
 Dv3 = 0 mL/min
 Dv2 = 100 mL/min
SVT
 Dv1 = 4660 mL/min
Ø4
Y
0mmHg
 Dv4 = 100 mL/min
I
Ø4
Ø8
Ø8
Ø4
Y
I
Ø9.5
 P2 = 92 mmHg
MOHR CLAMP
Ø9.5
 P1 = 83 mmHg
 Dv5 = 4560 mL/min
 P5 = 40 mmHg
Adjustment of arterial flow regime: iterations on total pump flow and Mohr clamp setting
Arterial regime (Flow & Pressure): brachial artery, with Ø4 mm instead of Ø5 mm
Dv2 = 100 mL/min ✓
P = 92 mmHg (i.e., ΔP A/V = 92 mmHg) ✓
Appendix 1
1

## Slide 2
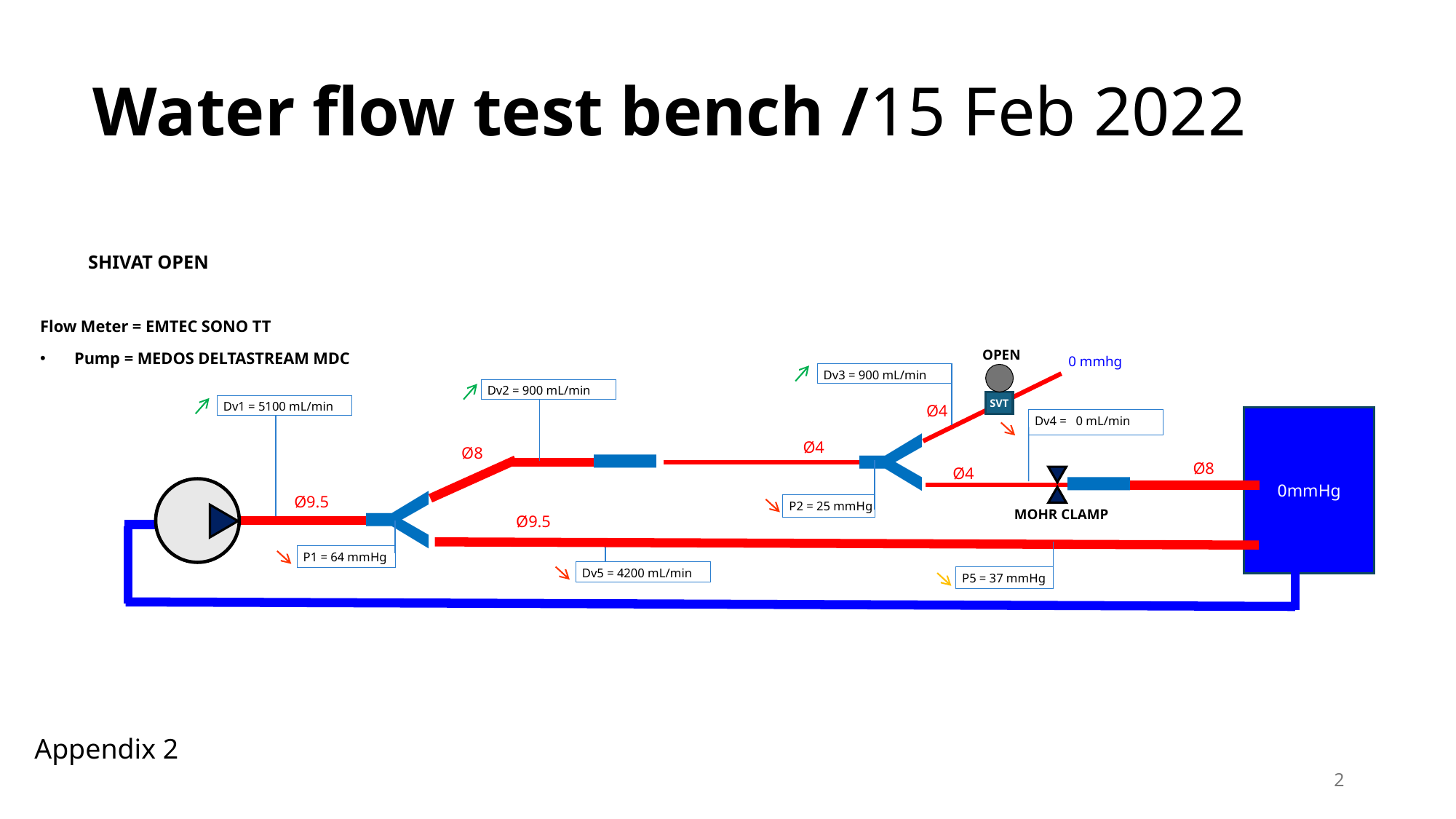

# Water flow test bench /15 Feb 2022
SHIVAT OPEN
Flow Meter = EMTEC SONO TT
Pump = MEDOS DELTASTREAM MDC
OPEN
0 mmhg
 Dv3 = 900 mL/min
 Dv2 = 900 mL/min
SVT
 Dv1 = 5100 mL/min
Ø4
Y
0mmHg
 Dv4 = 0 mL/min
I
Ø4
Ø8
Ø8
Ø4
Y
I
Ø9.5
 P2 = 25 mmHg
MOHR CLAMP
Ø9.5
 P1 = 64 mmHg
 Dv5 = 4200 mL/min
 P5 = 37 mmHg
Appendix 2
2

## Slide 3
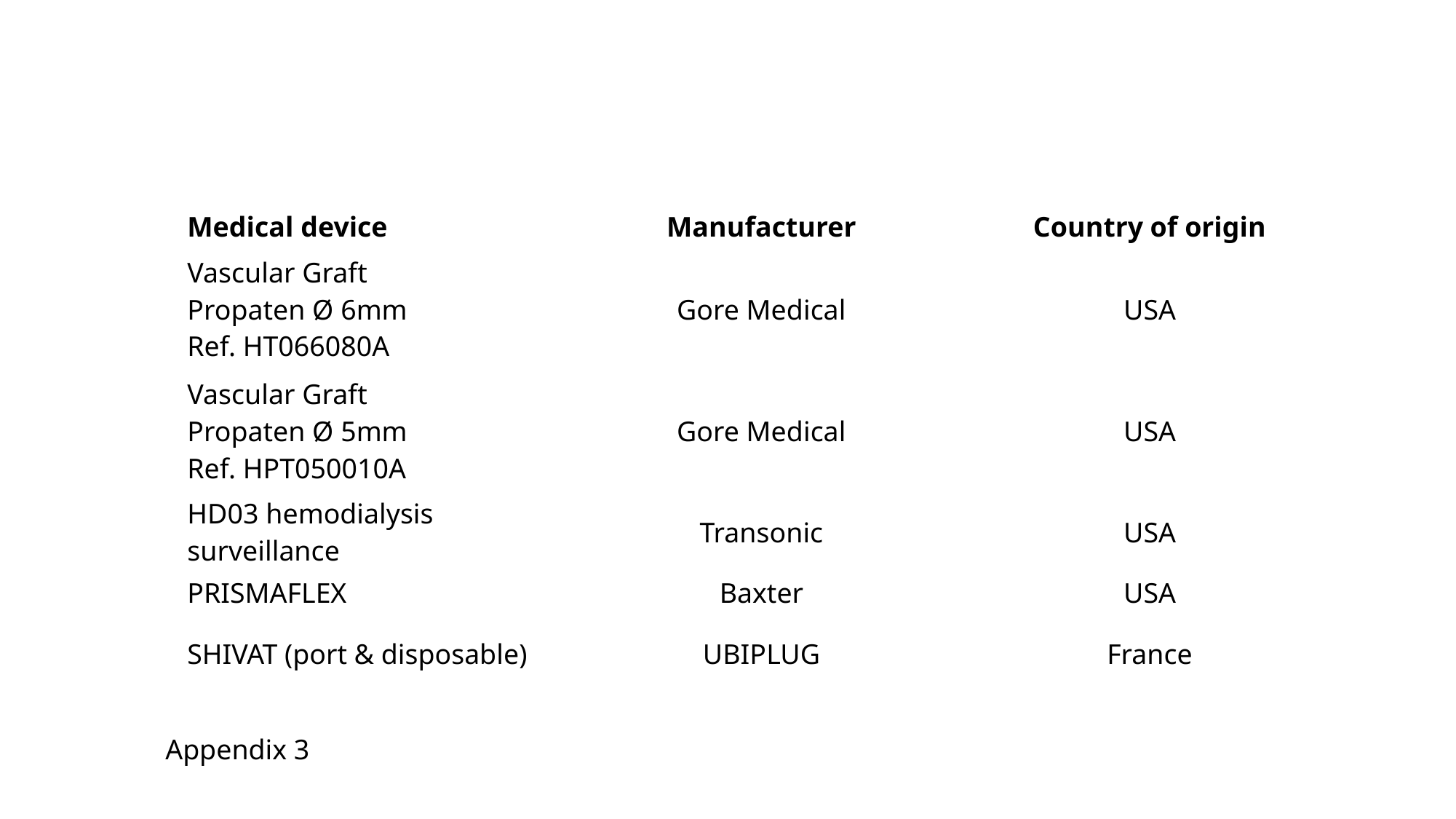

| Medical device | Manufacturer | Country of origin |
| --- | --- | --- |
| Vascular Graft Propaten Ø 6mm Ref. HT066080A | Gore Medical | USA |
| Vascular Graft Propaten Ø 5mm Ref. HPT050010A | Gore Medical | USA |
| HD03 hemodialysis surveillance | Transonic | USA |
| PRISMAFLEX | Baxter | USA |
| SHIVAT (port & disposable) | UBIPLUG | France |
Appendix 3
